# Magnesium, vitamin D status and mortality: results from US National Health and Nutrition Examination Survey (NHANES) 2001 to 2006 and NHANES III

**DOI:** 10.1186/1741-7015-11-187

**Published:** 2013-08-27

**Authors:** Xinqing Deng, Yiqing Song, JoAnn E Manson, Lisa B Signorello, Shumin M Zhang, Martha J Shrubsole, Reid M Ness, Douglas L Seidner, Qi Dai

**Affiliations:** 1 Department of Medicine, Division of Epidemiology, Vanderbilt University School of Medicine, Nashville, TN 37203, USA; 2 Division of Preventive Medicine, Brigham and Women’s Hospital, Harvard Medical School, Boston, MA 02115, USA; 3 Department of Epidemiology, Harvard School of Public Health, Boston, MA 02115, USA; 4 Department of Medicine, Division of Gastroenterology, Vanderbilt School of Medicine, Nashville, TN 37232, USA; 5 Institute for Medicine and Public Health, Vanderbilt University Medical Center, Nashville, TN 37203, USA

**Keywords:** Magnesium intake, Serum 25-hydroxyvitamin D levels, Vitamin D insufficiency, Vitamin D deficiency, Parathyroid hormone, Mortality, Colorectal cancer, Cardiovascular diseases

## Abstract

**Background:**

Magnesium plays an essential role in the synthesis and metabolism of vitamin D and magnesium supplementation substantially reversed the resistance to vitamin D treatment in patients with magnesium-dependent vitamin-D-resistant rickets. We hypothesized that dietary magnesium alone, particularly its interaction with vitamin D intake, contributes to serum 25-hydroxyvitamin D (25(OH)D) levels, and the associations between serum 25(OH)D and risk of mortality may be modified by magnesium intake level.

**Methods:**

We tested these novel hypotheses utilizing data from the National Health and Nutrition Examination Survey (NHANES) 2001 to 2006, a population-based cross-sectional study, and the NHANES III cohort, a population-based cohort study. Serum 25(OH)D was used to define vitamin D status. Mortality outcomes in the NHANES III cohort were determined by using probabilistic linkage with the National Death Index (NDI).

**Results:**

High intake of total, dietary or supplemental magnesium was independently associated with significantly reduced risks of vitamin D deficiency and insufficiency respectively. Intake of magnesium significantly interacted with intake of vitamin D in relation to risk of both vitamin D deficiency and insufficiency. Additionally, the inverse association between total magnesium intake and vitamin D insufficiency primarily appeared among populations at high risk of vitamin D insufficiency. Furthermore, the associations of serum 25(OH)D with mortality, particularly due to cardiovascular disease (CVD) and colorectal cancer, were modified by magnesium intake, and the inverse associations were primarily present among those with magnesium intake above the median.

**Conclusions:**

Our preliminary findings indicate it is possible that magnesium intake alone or its interaction with vitamin D intake may contribute to vitamin D status. The associations between serum 25(OH)D and risk of mortality may be modified by the intake level of magnesium. Future studies, including cohort studies and clinical trials, are necessary to confirm the findings.

## Background

Vitamin D deficiency causes rickets among children and osteomalacia in adults [1]. Many epidemiologic studies suggest that low vitamin D status may also be associated with risk of non-skeletal chronic diseases, such as, all-cause mortality [2-4], type 2 diabetes [5-7], cardiovascular diseases (CVD) [8,9], and colorectal cancer [10-12]. However, findings have not been entirely consistent [13-16]. Large-scale clinical trials of vitamin D supplementation are ongoing [13,14,17]. Despite food fortification and dietary supplementation, some studies have observed that low vitamin D status is still relatively common in the US [18] while a large portion of the interperson variation in serum 25-hydroxyvitamin D (25(OH)D) levels is unexplained [19,20].

**Figure 1:**
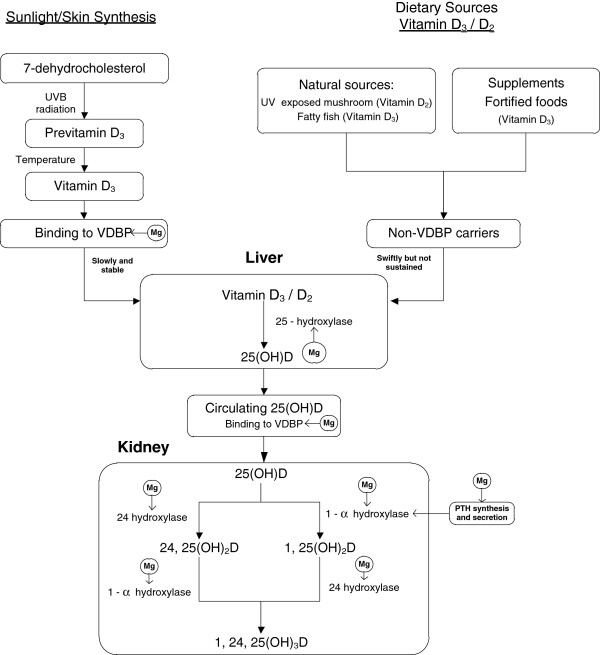
**Magnesium and metabolism of vitamin D.** PTH, parathyroid hormone; UVB, ultraviolet B; VDBP, vitamin D binding protein.

Magnesium, the second most abundant intracellular cation, plays a critical role in the synthesis and metabolism of parathyroid hormone (PTH) and vitamin D [21-23]. Previous studies have shown that the activities of three major enzymes determining 25(OH)D level [22-25] and vitamin D binding protein [23] are magnesium dependent (Figure 1). Magnesium deficiency, which leads to reduced 1,25(OH)_2_ vitamin D and impaired PTH response [23], has been implicated in ‘magnesium-dependent vitamin-D-resistant rickets’ [21]. Magnesium supplementation substantially reversed the resistance to vitamin D treatment [21]. Interestingly, a study conducted among osteoporosis patients showed much higher prevalence rates of magnesium deficiency or insufficiency among people with insufficient 25(OH)D than those with sufficient 25(OH)D [26]. Two small clinical trials of magnesium-deficient patients [23,27] found that magnesium infusion alone led to a non-significant increase in 1,25(OH)_2_D and 25(OH)D [23] whereas magnesium infusion plus oral vitamin D substantially increased both serum 25(OH)D and 1,25(OH)_2_D [27]. These findings suggest a potential interaction between vitamin D and magnesium treatments and a possible moderate effect of magnesium on 25(OH)D status.

We hypothesize that magnesium intake alone and particularly its interaction with vitamin D intake contribute to serum 25(OH)D status and tested this novel hypothesis utilizing data from the National Health and Nutrition Examination Survey (NHANES) 2001 to 2006. Furthermore, previous studies reported that serum 25(OHD) concentrations were associated with reduced risks of total mortality, particularly mortality due to colorectal cancer [28] and CVD [4,29]. We hypothesize that the inverse associations between serum 25(OH)D and risk of mortality are modified by intake level of magnesium and tested this hypothesis using the NHANES III cohort.

## Methods

### Participants

The 2001 to 2006 NHANES and the NHANES III were reviewed and approved by the National Center for Health Statistics (NCHS) Institutional Review Board (IRB) (Hyattsville, MD, USA). A detailed description of the study design has been published elsewhere [30,31]. To investigate if dietary magnesium alone and its interaction with vitamin D intake contribute to serum 25(OH)D levels, we utilized data from the NHANES 2001 to 2006, which was conducted during years 2001 to 2006 by the NCHS of the Centers for Disease Control and Prevention (CDC) (Atlanta, GA, USA). This is a nationally representative and recent sample among the civilian, non-institutionalized US population. Thus, the status of serum 25(OH)D represents the current vitamin D status of US population. Furthermore, serum PTH data are available only in the NHANES conducted during 2003 to 2006. We limited our study population to 12257 participants aged ≥20 years with serum 25(OH)D, reliable dietary data, and negative for pregnancy test. However, no follow-up of mortality outcomes were conducted for NHANES 2001 to 2006. The mortality outcomes have been prospectively followed in the NHANES III cohort for which baseline data and blood collection were conducted during 1988 to 1994 among a nationally representative sample of US adults. Included in the NHANES III cohort were those aged ≥17 years with 25(OH)D measurements (n = 16819). The participants were followed for mortality status from baseline until December 31, 2006. Thus, we used the NHANES III cohort to examine the modifying effects of magnesium intake on the associations between serum 25(OH)D and risk of mortality. Although the NHANES III cohort is suitable for the investigation of the associations between serum 25(OH)D and mortality outcomes, no serum PTH data are available. NHANES III was approved by the National Center for Health Statistics (NCHS) Institutional Review Board (IRB) and documented consent was obtained from participants. The NCHS IRB (before 2003)/and the NCHS Research Ethics Review Board (ERB) (year 2003 and after) approved the NHANES 2001 to 2006 (protocol #98-12 for the NHANES 2001 to 2004 and protocol #2005-06 for the NHANES 2005 to 2006).

### Blood sample collection and measures of serum 25(OH)D

Over 88% of the 2001 to 2006 NHANES participants and about 90% of the NHANES III participants donated a spot blood sample. Measurements of serum 25(OH)D were performed at the NCH, CDC, Atlanta, GA using a radioimmunoassay (RIA) kit (Diasorin Inc., Stillwater MN, USA) [32] and the coefficients of variations (CV) were 13% to 19% in the NHANES III and 10% to 13% in the NHANES 2001 to 2006 [29]. Serum PTH concentrations were measured using the ECL/Origen electrochemiluminescent process (Elecsys 1010 system, Roche Diagnostics, Indianapolis, IN, USA) and CV was less than 10%. Data were available only for the NHANES 2003 to 2006.

### Outcomes

We have utilized the cut-off points as defined in the recently issued Institute of Medicine (IOM) report to define vitamin D deficiency and insufficiency [33]. We classified the participants in the NHANES 2001 to 2006 into the following three categories: (1) participants who had IOM recommended vitamin D level (serum 25(OH)D ≥20 ng/ml (50 nmol/l)); (2) participants who had insufficient vitamin D (serum 25(OH)D ≥12 ng/ml (30 nmol/l) but below 20 ng/ml (50 nmol/l)); and (3) participants who had vitamin D deficiency (serum 25(OH)D <12 ng/ml (30 nmol/l)). In addition to Institute of Medicine’s recommendation, the other reason we used 20 ng/ml as the cut-off point is that the median value of serum 25(OH)D for the NHANES 2001 to 2006 was 21.0 ng/mg, which is very close to 20 ng/ml.

In the NHANES III cohort, mortality outcomes were determined by using probabilistic linkage with the National Death Index (NDI). For mortality due to CVD or colorectal cancer, participants who died of other diseases or were not known deceased, were censored at the date of death or December 31, 2006, whichever was earlier.

### Nutrient intake assessments

Daily dietary intake data were obtained from 24-h dietary recalls and 30-day supplement interviews, which are described in detail elsewhere [34]. Only one 24-h recall was conducted in NHANES III and from 2001 to 2002. To keep intake information consistent through the study period only the first dietary recall for all subjects was utilized in the present analysis. Only dietary recall data with a status of ‘reliable’ were used in the analysis. Supplemental doses and intakes of magnesium, vitamin D, and calcium were obtained from the response to a dietary supplement questionnaire.

### Statistical analysis

We performed statistical analyses using PROC Survey in SAS 9.2 software (SAS Institute, Cary, NC, USA) to estimate variance after incorporating the weights for the sample population in NHANES.

Covariates were compared among three groups with differing vitamin D status (Table 1). Any covariate that significantly differed among groups was considered as a potential confounding factor. In particular, those confounders that altered the estimate of association by 10% or more were retained in the final models (see footnotes to Tables 2 and 3). Other factors, such as use of phosphorus, potassium, and retinol, did not materially alter the risk estimates. Multivariate adjusted odds ratios (ORs) and 95% confidence intervals (CIs) for vitamin D deficiency and insufficiency were calculated. Stratified analyses by potential effect modifiers and tests for multiplicative interactions using the Wald test were conducted.

**Table 1:** Selected characteristics of the study participants by serum 25(OH)D levels, National Health and Nutrition Examination Survey (NHANES) 2001 to 2006

**Characters**^**a**^	**Normal, n = 6962**	**Insufficiency, n = 3620**	**Deficiency, n = 1575**	***P *value**^**b**^
Serum 25(OH)D (ng/ml), mean (SE)	28.5 (0.2)	16.1 (0.2)	8.8 (0.1)	<0.001
Age (years), mean (SE)	47.0 (0.4)	46.7 (0.4)	46.2 (0.6)	<0.001
Male, n (%)	3711 (51.2)	1833 (47.7)	696 (38.4)	<0.001
Body mass index (kg/m^2^), mean (SE)	27.3 (0.1)	29.6 (0.2)	31.5 (0.4)	<0.001
Month of blood collection, n (%)				
1 November to 30 April (winter)	2,688 (34.4)	1,980 (50.7)	999 (57.6)	<0.001
1 May to 31 October (summer)	4,274 (65.6)	1,640 (49.3)	676 (42.4)	
Education, n (%)				
Less than high school	1,694 (14.8)	1,194 (21.8)	621 (28.5)	<0.001
High school	1,729 (25.3)	872 (26.3)	384 (25.4)	
Greater than high school	3,535 (59.9)	1,550 (51.9)	667 (46.1)	
Race/ethnicity, n (%)				
Non-Hispanic Caucasian	4,812 (84.5)	1,390 (59.5)	316 (33.9)	<0.001
Non-Hispanic Black	558 (3.5)	965 (17.0)	906 (44.8)	
Hispanic	1,394 (8.6)	1,091 (16.5)	394 (14.1)	
Others	198 (3.4)	174 (7.1)	59 (7.1)	
Family poverty income ratio, n (%)				
<1	929 (9.7)	661 (15.6)	358 (20.0)	<0.001
1 to 4	4,181 (61.5)	2,230 (63.6)	1,031 (65.7)	
≥5	1,507 (28.8)	516 (20.8)	181 (14.3)	
Smoker, n (%)				
Non-smoker	3,407 (49.5)	1,856 (50.0)	869 (50.0)	<0.001
Former smoker	1,914 (24.8)	833 (21.0)	303 (16.3)	
Current smoker	1,637 (25.7)	926 (29.0)	500 (33.8)	
Alcohol user, n (%)				
Non-drinker	784 (15.0)	555 (21.5)	293 (24.2)	<0.001
Former drinker	559 (10.3)	382 (14.3)	187 (14.1)	
Current drinker	3,139 (74.8)	1,452 (64.2)	639 (61.7)	
Physical activity during the day, n (%)				
Sit and not walk about very much	1,512 (21.2)	960 (27.0)	541 (32.9)	<0.001
Walk a lot	3,584 (50.4)	1,904 (50.5)	857 (49.2)	
Light work	1,276 (20.5)	497 (15.2)	202 (13.9)	
Heavy work	582 (8.0)	254 (7.3)	73 (4.0)	
Leisure time physical activity (Metabolic equivalent/h/week), mean (SE)	1,292.4 (52.2)	788.5 (55.6)	674.2 (53.1)	<0.001
Users of supplement containing vitamin D (%)	3,258 (47.2)	1,018 (30.4)	202 (12.5)	<0.001
Users of supplement containing Ca (%)	3,860 (57.1)	1,380 (42.6)	351 (22.3)	<0.001
Users of supplement containing Mg (%)	3,040 (45.1)	1,072 (32.0)	235 (14.9)	<0.001
Energy-adjusted nutrient intakes^c^, mean (SE)				
Protein (g/day)	82.0 (0.5)	80.1 (0.7)	77.8 (1.3)	<0.01
Carbohydrate (g/day)	258.5 (1.4)	262.4 (1.7)	261.6 (2.9)	0.135
Fat (g/day)	80.7 (0.5)	80.3 (0.6)	81.2 (0.9)	0.93
Calcium (mg/day)	929.0 (8.4)	805.2 (9.1)	690.3 (13.6)	<0.001
Magnesium (mg/day)	293.1 (2.2)	266.6 (2.9)	252.4 (4.2)	<0.001
Vitamin D (IU/day)	196 (8.0)	160 (8.0)	116 (8.0)	<0.001
Total calcium (mg/day)^d^	1,168.8 (12.6)	935.4 (12.3)	748.5 (15.9)	<0.001
Total magnesium (mg/day)^d^	344.2 (3.5)	297.3 (3.2)	262.4 (4.4)	<0.001
Total vitamin D (IU/day)^d^	376 (8.0)	276 (8.0)	152 (8.0)	<0.001

^a^Values are present as weighted means (SE) and unweighted frequencies (weighted percentages, %).

^b^Rao-Scott χ^2^ test for categorical data and analysis of variance (ANOVA) test for continuous variables.

^c^Values are energy-adjusted means (SE).

^d^Total calcium, magnesium and vitamin D intake from foods and supplements.

**Table 2:** **Multivariate-adjusted odds ratios (95% CIs)**^**a **^**for associations between total intakes of vitamin D and magnesium and risk of vitamin D deficiency and insufficiency, National Health and Nutrition Examination Survey (NHANES) 2001 to 2006**

**Nutrient intakes**	**Controls, N**	**Deficiency**		**Insufficiency**	
		**N**	**OR (95% CI)**	**N**	**OR (95% CI)**
Total vitamin D intake (IU/day)					
Q1 <114	1,741	1,005	1.00	1,450	1.00
Q2 114 to 307	1,740	446	0.55 (0.40 to 0.75)	1,086	0.75 (0.62 to 0.91)
Q3 308 to 539	1,741	152	0.22 (0.15 to 0.34)	665	0.55 (0.44 to 0.69)
Q4 ≥540	1,740	72	0.10 (0.06 to 0.17)	419	0.37 (0.27 to 0.51)
* P* for trend			<0.001		<0.001
Dietary vitamin D intake (IU/day)					
Q1 <57.8	1,741	764	1.00	1,183	1.00
Q2 57.8 to 137	1,740	468	0.71 (0.55 to 0.91)	997	0.86 (0.71 to 1.05)
Q3 138 to 261	1,741	279	0.54 (0.40 to 0.72)	807	0.72 (0.59 to 0.88)
Q4 ≥262	1,740	164	0.29 (0.20 to 0.41)	633	0.69 (0.56 to 0.86)
*P* for trend			<0.001		<0.001
Supplemental vitamin D intake (IU/day)					
Non-user	3,704	1,473	1.00	2,602	1.00
1 to 239	809	93	0.32 (0.20 to 0.50)	377	0.84 (0.68 to 1.03)
240 to 399	331	19	0.29 (0.13 to 0.64)	109	0.57 (0.38 to 0.87)
≥400	2,118	90	0.19 (0.11 to 0.33)	532	0.40 (0.29 to 0.53)
*P* for trend			<0.001		<0.001
Total magnesium intake (mg/day)					
Q1 <225	1,749	876	1.00	1,362	1.00
Q2 225 to 310	1,733	392	0.67 (0.50 to 0.90)	907	0.78 (0.61 to 1.00)
Q3 311 to 419	1,737	245	0.43 (0.30 to 0.63)	758	0.73 (0.59 to 0.89)
Q4 ≥420	1,743	162	0.34 (0.21 to 0.56)	593	0.62 (0.46 to 0.82)
*P* for trend			<0.001		<0.001
Dietary magnesium intake (mg/day)					
Q1 <195	1,754	726	1.00	1,255	1.00
Q2 195 to 268	1,732	432	0.75 (0.58 to 0.97)	893	0.75 (0.62 to 0.90)
Q3 269 to 363	1,741	297	0.68 (0.47 to 0.99)	777	0.69 (0.55 to 0.87)
Q4 ≥364	1,735	220	0.43 (0.26 to 0.70)	695	0.61 (0.45 to 0.83)
*P* for trend			0.002		0.002
Supplemental magnesium intake (mg/day)					
Non-user	3,922	1,440	1.00	2,548	1.00
1 to 49	669	109	0.53 (0.34 to 0.81)	334	1.14 (0.91 to 1.43)
50 to 99	506	43	0.35 (0.17 to 0.71)	205	0.80 (0.53 to 1.22)
≥100	1,865	83	0.30 (0.17 to 0.52)	533	0.68 (0.52 to 0.88)
*P* for trend			<0.001		0.008

^a^Survey logistic regression models used to estimate odds ratios and 95% confidence intervals. Models adjusted for age, sex, body mass index (BMI), educational attainment, race, household income, smoking status, alcohol use, physical activity, month of blood collection, dietary intakes of total energy and total calcium intake. Models were also adjusted for total intake of vitamin D or magnesium mutually.

**Table 3:** **Odds ratios (95% CI)**^**a **^**for vitamin D deficiency and insufficiency stratified by the median of vitamin D intake or season, National Health and Nutrition Examination Survey (NHANES) 2001 to 2006**

**Magnesium intake, mg/day**	**Controls, N**	**Deficiency**		**Insufficiency**	
		**N**	**OR (95% CI)**	**N**	**OR (95% CI)**
Vitamin D intake <308 (IU/day)					
Q1 <225	1,359	834	1.00	1,218	1.00
Q2 225 to 310	940	328	0.79 (0.58 to 1.07)	650	0.89 (0.69 to 1.15)
Q3 311 to 419	697	184	0.64 (0.46 to 0.91)	426	0.78 (0.63 to 1.08)
Q4 ≥420	485	105	0.58 (0.35 to 0.97)	242	0.71 (0.47 to 1.07)
*P* for trend			0.01		<0.05
Vitamin D intake ≥308 (IU/day)					
Q1 <225	390	42	1.00	144	1.00
Q2 225 to 310	793	64	1.09 (0.47 to 2.55)	257	0.94 (0.62 to 1.42)
Q3 311 to 419	1,040	61	0.76 (0.30 to 1.88)	332	1.19 (0.80 to 1.77)
Q4 ≥420	1,258	57	0.71 (0.24 to 2.11)	351	0.96 (0.60 to 1.55)
*P* for trend			0.97		0.67
*P* for interaction			<0.001		<0.001
Non-vitamin D supplement user					
Q1 <225	1,292	812	1.00	1,160	1.00
Q2 225 to 310	958	332	0.81 (0.57 to 1.14)	651	0.91 (0.70 to 1.18)
Q3 311 to 419	793	205	0.59 (0.40 to 0.88)	462	0.80 (0.63 to 1.02)
Q4 ≥420	661	124	0.60 (0.35 to 1.00)	329	0.81 (0.55 to 1.19)
*P* for trend			0.02		0.18
Vitamin D supplement user					
Q1 <225	457	64	1.00	202	1.00
Q2 225 to 310	775	60	1.23 (0.59 to 2.56)	256	0.80 (0.53 to 1.20)
Q3 311 to 419	944	40	1.05 (0.41 to 1.68)	296	0.96 (0.65 to 1.41)
Q4 ≥420	1,082	38	0.91 (0.35 to 2.36)	264	0.73 (0.49 to 1.10)
*P* for trend			0.56		0.40
*P* for interaction			0.47		0.37
Winter and Southern latitude					
Q1 <225	664	493	1.00	726	1.00
Q2 225 to 310	691	239	0.63 (0.47 to 0.85)	495	0.73 (0.57 to 0.92)
Q3 311 to 419	653	156	0.49 (0.30 to 0.78)	422	0.89 (0.74 to 1.07)
Q4 ≥420	680	111	0.54 (0.28 to 1.02)	337	0.57 (0.41 to 0.78)
*P* for trend			0.03		<0.01
Summer and Northern latitude					
Q1 <225	1,085	383	1.00	636	1.00
Q2 225 to 310	1,042	153	0.95 (0.64 to 1.41)	412	0.91 (0.66 to 1.26)
Q3 311 to 419	1,084	89	0.61 (0.43 to 0.86)	336	0.73 (0.56 to 0.96)
Q4 ≥420	1,063	51	0.33 (0.20 to 0.55)	256	0.85 (0.61 to 1.19)
*P* for trend			0.001		0.35
*P* for interaction			0.55		0.21
Age <50 (years)					
Q1 <225	862	465	1.00	726	1.00
Q2 225 to 310	821	209	0.74 (0.49 to 1.11)	495	0.83 (0.60 to 1.16)
Q3 311 to 419	831	150	0.63 (0.35 to 1.13)	422	0.84 (0.62 to 1.13)
Q4 ≥420	892	110	0.67 (0.32 to 1.42)	337	0.73 (0.50 to 1.08)
*P* for trend			0.26		0.15
Age ≥50 (years)					
Q1 <225	1,085	383	1.00	636	1.00
Q2 225 to 310	1,042	153	0.85 (0.50 to 1.37)	412	0.86 (0.63 to 1.16)
Q3 311 to 419	1,084	89	0.43 (0.24 to 0.77)	336	0.79 (0.57 to 1.10)
Q4 ≥420	1,063	51	0.24 (0.11 to 0.49)	256	0.71 (0.46 to 1.10)
*P* for trend			<0.01		0.81
*P* for interaction			0.07		0.41
Male					
Q1 <225	712	283	1.00	519	1.00
Q2 225 to 310	854	164	0.64 (0.41 to 1.00)	466	1.12 (0.78 to 1.61)
Q3 311 to 419	995	143	0.39 (0.21 to 0.74)	439	0.90 (0.65 to 1.26)
Q4 ≥420	1,150	106	0.33 (0.16 to 0.69)	409	0.95 (0.59 to 1.52)
*P* for trend			0.001		0.54
Female					
Q1 <225	1,037	593	1.00	843	1.00
Q2 225 to 310	879	228	0.85 (0.61 to 1.21)	441	0.71 (0.54 to 0.93)
Q3 311 to 419	742	102	0.63 (0.37 to 1.05)	319	0.83 (0.64 to 1.07)
Q4 ≥420	593	56	0.47 (0.25 to 0.87)	184	0.60 (0.43 to 0.82)
*P* for trend			0.16		0.14
*P* for interaction			0.11		0.17
Non-Hispanic Black					
Q1 <225	181	530	1.00	442	1.00
Q2 225 to 310	163	181	0.51 (0.34 to 0.76)	229	0.74 (0.53 to 1.03)
Q3 311 to 419	110	115	0.44 (0.26 to 0.74)	175	0.53 (0.33 to 0.88)
Q4 ≥420	104	81	0.43 (0.27 to 0.68)	119	0.64 (0.39 to 1.04)
*P* for trend			<0.001		<0.05
Non-Hispanic Caucasian and others					
Q1 <225	1,568	346	1.00	920	1.00
Q2 225 to 310	1,570	212	0.91 (0.65 to 1.28)	678	0.86 (0.67 to 1.10)
Q3 311 to 419	1,627	130	0.66 (0.45 to 0.97)	583	0.87 (0.67 to 1.10)
Q4 ≥420	1,639	81	0.52 (0.29 to 0.95)	474	0.76 (0.58 to 0.99)
*P* for trend			<0.05		0.27
*P* for interaction			0.305		0.82
BMI <25 (kg/m^2^)					
Q1 <225	631	172	1.00	336	1.00
Q2 225 to 310	611	90	1.04 (0.51 to 2.12)	339	0.92 (0.61 to 1.40)
Q3 311 to 419	606	58	0.84 (0.40 to 1.76)	189	1.00 (0.70 to 1.44)
Q4 ≥420	608	41	0.51 (0.23 to 1.15)	162	0.77 (0.47 to 1.27)
*P* for trend			0.13		0.45
BMI ≥25 to <30 (kg/m^2^)					
Q1 <225	605	232	1.00	448	1.00
Q2 225 to 310	632	106	0.71 (0.43 to 1.15)	316	0.77 (0.51 to 1.17)
Q3 311 to 419	661	75	0.50 (0.29 to 0.86)	266	0.63 (0.50 to 0.79)
Q4 ≥420	677	46	0.47 (0.22 to 1.01)	213	0.73 (0.48 to 1.13)
*P* for trend			0.02		0.06
BMI ≥30 (kg/m^2^)					
Q1 <225	463	440	1.00	532	1.00
Q2 225 to 310	457	177	0.63 (0.35 to 1.14)	341	0.82 (0.63 to 1.06)
Q3 311 to 419	441	108	0.46 (0.24 to 0.88)	288	0.83 (0.59 to 1.15)
Q4 ≥420	424	69	0.42 (0.19 to 0.96)	202	0.63 (0.44 to 0.91)
*P* for trend			0.02		0.02
*P* for interaction			0.23		0.13
PTH <32 (pg/ml)					
Q1 <225	322	63	1.00	179	1.00
Q2 225 to 310	369	39	0.42 (0.18 to 1.02)	109	0.72 (0.42 to 1.24)
Q3 311 to 419	363	21	0.17 (0.07 to 0.43)	107	0.77 (0.47 to 1.28)
Q4 ≥420	392	19	0.22 (0.07 to 0.73)	100	0.74 (0.38 to 1.42)
*P* for trend			<0.01		0.46
PTH ≥32 to <46 (pg/ml)					
Q1 <225	378	136	1.00	271	1.00
Q2 225 to 310	382	66	1.20 (0.66 to 2.21)	195	0.94 (0.73 to 1.22)
Q3 311 to 419	406	54	1.19 (0.53 to 2.66)	181	0.85 (0.59 to 1.23)
Q4 ≥420	414	33	1.11 (0.36 to 3.42)	131	0.79 (0.55 to 1.15)
*P* for trend			0.85		0.24
PTH ≥46 (pg/ml)					
Q1 <225	390	375	1.00	447	1.00
Q2 225 to 310	382	157	0.73 (0.48 to 1.10)	297	0.93 (0.68 to 1.28)
Q3 311 to 419	382	95	0.52 (0.28 to 0.95)	209	0.78 (0.60 to 1.01)
Q4 ≥420	365	50	0.28 (0.14 to 0.53)	159	0.57 (0.35 to 0.92)
*P* for trend			0.01		0.41
*P* for interaction			0.73		0.53

Parathyroid hormone (PTH) laboratory results were only available from the NHANES 2003 to 2006.

^a^Survey logistic regression models used to estimate odds ratios and 95% confidence intervals. Models were adjusted for age, sex, body mass index (BMI), educational attainment, race, household income, smoking status, alcohol use, physical activity, month of blood collection, dietary intakes of total energy, total intake of calcium and vitamin D.

Hazard ratios (HR) were estimated in Cox proportional hazard regression models to examine whether the associations between serum 25(OH)D and risk of mortality differed by intake level of total magnesium, using the data from the NHANES III cohort. The same cut-off points for serum 25(OH)D were used as those in the published report [28]. We adjusted for the same confounding factors as we did for vitamin D deficiency and insufficiency analyses. All reported *P* values were two-sided. *P* values of <0.05 were considered statistically significant in all analyses.

In Table 4, we present the associations between serum 25(OH)D and risk of mortality within two strata (<264 mg/day and ≥264 mg/day) of magnesium intake. However, this is only for data presentation purpose. In statistical modeling, we have used continuous variables for both serum 25(OH)D and intakes of magnesium in the tests for interactions between serum 25(OH)D and intakes of magnesium in relation to total mortality, mortality due to cardiovascular disease and mortality due to colorectal cancer. We chose 264 mg/day as the cut-off point presented in Table 4 based on both statistical power consideration and biological support. We have a limited sample size for colorectal cancer mortality outcome. By using a median magnesium intake of 264 mg/day, we could maximize the sample size, and, thus, the power to detect association between vitamin D levels and mortality outcomes in both strata. Furthermore, 264 mg/day is very close to the 265 mg/day, the estimated average requirements (EAR) for women and so it gave us the opportunity to evaluate the relationship to the EAR [35].

**Table 4:** **Hazard ratios (HRs)**^**a **^**and 95% CIs for total mortality in the National Health and Nutrition Examination Survey (NHANES) III (1988 to 2006)**

**Magnesium intake (mg/day)**		**Serum vitamin D level (ng/ml)**				***P *for trend**
		**<20**	**20 to 31**	**32 to 39**	≥**40**	
Total mortality						
Total	Cases	1,274	1,547	558	324	
	HR (95% CI)	1.00	0.83 (0.74 to 0.94)	0.80 (0.69 to 0.93)	0.79 (0.66 to 0.93)	<0.01
<264	Cases	848	786	249	143	
	HR (95% CI)	1.00	0.89 (0.74 to 1.08)	0.87 (0.69 to 1.08)	0.87 (0.64 to 1.20)	0.23
≥264	Cases	426	761	309	181	
	HR (95% CI)	1.00	0.77 (0.64 to 0.92)	0.73 (0.58 to 0.93)	0.70 (0.57 to 0.88)	<0.01
*P* for interaction						0.96
Cardiovascular mortality						
Total	Cases	574	656	253	132	
	HR (95% CI)	1.00	0.73 (0.62 to 0.86)	0.77 (0.61 to 0.99)	0.73 (0.56 to 0.97)	0.04
<264	Cases	386	346	112	57	
	HR (95% CI)	1.00	0.83 (0.66 to 1.04)	0.88 (0.61 to 1.28)	0.91 (0.61 to 1.35)	0.45
≥264	Cases	188	310	141	75	
	HR (95% CI)	1.00	0.59 (0.44 to 0.80)	0.65 (0.44 to 0.95)	0.57 (0.38 to 0.87)	<0.05
*P* for interaction						0.03
Colorectal cancer mortality						
Total	Cases	32	34	12	8	
	HR (95% CI)	1.00	0.84 (0.36 to 2.00)	0.80 (0.31 to 2.05)	0.50 (0.15 to 1.73)	0.31
<264	Cases	19	17	3	3	
	HR (95% CI)	1.00	1.17 (0.39 to 3.56)	0.79 (0.17 to 3.75)	0.85 (0.17 to 4.12)	0.73
≥264	Cases	13	17	9	5	
	HR (95% CI)	1.00	0.55 (0.16 to 1.85)	0.67 (0.17 to 2.64)	0.28 (0.03 to 2.25)	0.29
*P* for interaction						0.15

^a^A Cox’s proportional hazards model was performed with the SURVEYPHREG procedure to estimate odds ratios and 95% confidence intervals. Models were adjusted for age, race, sex, BMI, education attainment, household income, smoking status, alcohol use, physical activity, month of blood collection, intakes of total energy, phosphorus, calcium and magnesium.

## Results

Selected demographic characteristics and potential confounding factors by the three categories of serum 25(OH)D status are shown in Table 1. Compared to the Estimate Average Requirements (EAR, intake levels for vitamin D (400 IU/day) and magnesium (330 mg/day) recommended by the US Food and Nutrition Board of the Institute of Medicine), the vitamin D normal group generally met the recommended intake levels for both vitamin D and magnesium whereas the average intake levels of these nutrients were significantly lower in the vitamin D-insufficient group and much lower among the vitamin D-deficient group.

After adjusting for confounding factors, the ORs (95% CI) for vitamin D deficiency and insufficiency were 0.10 (0.06 to 0.17) and 0.37 (0.27 to 0.51) comparing the highest quartile intake of total vitamin D versus the lowest (*P*_trend_ <0.001) (Table 2). The corresponding ORs (95% CI) were 0.34 (0.21 to 0.56) and 0.62 (0.46 to 0.82) for total magnesium intake (*P*_trend_ <0.001), respectively. In addition, we found that both dietary and supplemental intakes of vitamin D and magnesium were significantly inversely associated with risks of vitamin D deficiency and insufficiency.

In stratified analyses by intake of vitamin D and other factors related to vitamin D status (Table 3), we found intake of magnesium significantly interacted with intake of vitamin D in relation to both vitamin D deficiency (*P*_interaction_, <0.001) and insufficiency (*P*_interaction_, <0.001). The inverse association between magnesium intake and risk of vitamin D deficiency only appeared significant among those older than 50 years or with serum PTH level being in the highest or lowest tertile category. Meanwhile, the inverse associations between magnesium intake and risk of vitamin D insufficiency were statistically significant only among people at high risk of vitamin D insufficiency, such as those whose samples were collected during winter (at southern latitude), those with vitamin D intake below the median, women, non-Hispanic Blacks, obese individuals or those with the PTH levels in the highest tertile.

As reported previously, high levels of serum 25(OH)D were associated with a reduced risk of mortality due to all-cause CVD [4,29,36], and colorectal cancer [28]. We found that the inverse associations for higher serum 25(OH)D with risks of total mortality and mortality due to CVD were only statistically significant among those with magnesium intake above the median (Table 4). Although the test for interaction was not statistically significant for total mortality, it was statistically significant for mortality due to CVD (*P*_interaction_, 0.03). Sample size was small for mortality due to colorectal cancer. None of the associations, including the main association, were statistically significant. However, the association pattern in stratified analysis (*P*_interaction_, 0.15) was very similar to that for total mortality and mortality due to CVD.

## Discussion

Consistent with our hypothesis, we observed that high intake of total, dietary or supplemental magnesium was independently and significantly associated with reduced risks of both vitamin D deficiency and insufficiency. Furthermore, intake of magnesium significantly interacted with intakes of vitamin D in relation to both vitamin D deficiency and insufficiency. In the NHANES III cohort, a population-based prospective study, we found the inverse associations of serum 25(OH)D with mortality, particularly mortality due to CVD and colorectal cancer, were modified by magnesium intake, and the inverse associations were primarily present among those with magnesium intake above the median. In addition, we found the inverse association between magnesium intake and risk of vitamin D deficiency primarily occurred in those who had the highest or the lowest tertile of PTH level; while the inverse association between total magnesium intake and vitamin D insufficiency primarily appeared among populations at high risk of vitamin D insufficiency. To the best of our knowledge, this is the first study to examine the interaction between vitamin D and magnesium in association to mortality; and this is the first study to suggest a potential independent contribution of total magnesium intake and its interaction with vitamin D intake to vitamin D status in the general population.

Under normal physiologic conditions, 25(OH)D is derived primarily from endogenous synthesis via exposure of skin to sunlight because few natural foods contain vitamin D except by fortification or supplementation (see Figure 1). Vitamin D_3_ or D_2_ is transferred to the liver via vitamin D binding protein (VDBP) and converted to 25(OH)D by 25-hydroxylase and subsequently carried to the kidney by VDBP and converted to 1,25(OH)_2_D by 1α-hydroxylase enzyme. Both 25(OH)D and 1,25(OH)_2_D can be converted by 24-hydroxylase to the 24,25(OH)_2_D or 1,24,25(OH)_3_D, respectively [37]. Therefore, 25(OH)D levels are primarily determined by VDBP, 25-hydroxylase, 1α-hydroxylase and 24-hydroxylase activity, a fact that has recently been substantiated by a genome-wide association study [38]. Based on previous *in vitro* studies, magnesium status regulates both 1α-hydroxylase and 24-hydroxylase activity [22,24]. Previous studies indicated both VDBP [23] and 25-hydroxylase [25,39] might also be magnesium dependent. Therefore, magnesium would be expected to play an important role in 25(OH)D metabolism.

A previous clinical study found that parenteral magnesium treatment without vitamin D replacement in 23 magnesium-deficient patients led to a 12% rise in 25(OH)D and 30% increase in 1,25(OH)_2_D, but both changes were not statistically significant [23]. In a subsequent study of five magnesium-deficient patients, intramuscular treatment with magnesium alone also did not significantly increase 25(OH)D, but magnesium infusion together with pharmacological dose of 25(OH)D substantially increased both 25(OH)D and 1,25(OH)_2_D among patients with magnesium deficiency. One interpretation is that magnesium treatment does not affect 25(OH)D status [23,27]. However, we postulate that several factors may have contributed to the insignificant increase in 25(OH)D status. First, the subjects participating in these studies had low concentrations of 25(OH)D and 1,25(OH)_2_D as well as pre-vitamin D_3_ and vitamin D_3_ as a result of limited sunlight exposure, underlying disease and/or lack of oral supplementation. Therefore, concentrations of 25(OH)D and 1,25(OH)_2_D did not substantially increase during short-term magnesium repletion because pre-vitamin D3 was not available in sufficient amounts. Second, there was a modest increase in the conversion of 25(OH)D to 1,25(OH)_2_D and, thus, a reduction in 25(OH)D level was expected due to this conversion [22]. Finally, the sample size in these two studies was very small particularly if the direct effect of magnesium treatment on vitamin D status is only moderate.

We found that high magnesium intake was also associated with a reduced risk of vitamin D deficiency or insufficiency. We believe that this observation is the result of the interaction between various metabolic pathways that regulate 25(OH)D levels. Previous studies have shown that endogenously synthesized vitamin D_3_ is transferred almost completely by VDBP to liver and this transport is slow, leading to a more sustained plasma vitamin D_3,_ compared to that from supplementation of vitamin D, which is delivered to the liver by non-VDBP carriers in the plasma [40]. VDBP may also be an important determinant of serum 25(OH)D concentration, particularly when dietary intake of vitamin D is low. In the study by Rude *et al*., the concentration of VDBP was lower among 11 magnesium-deficient patients and significantly increased to normal after magnesium treatment without vitamin D supplementation [23]. Therefore, it is possible that an improvement in magnesium status leads to an increase in VDBP synthesis and, in turn, an elevated transport of vitamin D_3_ to the liver and 25(OH)D to the kidney.

The critical roles of magnesium in the synthesis of VDBP, PTH, 25(OH)D and 1,25(OH)_2_D may partially explain why the inverse associations between serum 25(OH)D and risk of total mortality and mortality due to colorectal cancer and CVD primarily existed among those with magnesium intake above the median. High magnesium may increase the availability of 1,25 (OH)2D through activating the synthesis of 25(OH)D and 1,25 (OH)2D and increasing the transfer to target tissues by elevating vitamin D binding protein (VDBP) (Figure 1). This explanation is also supported by the observation in previous clinical studies that magnesium supplementation substantially reversed the resistance to vitamin D treatment among magnesium-deficient patients [21].

Previous studies found PTH level was elevated when serum 25(OH)D was under 20 ng/ml [41]. In the current study, we found magnesium intake was associated with a reduced risk of vitamin D insufficiency or deficiency only among those in the highest or the lowest tertile of PTH. This finding is possible because magnesium plays an important role in PTH regulation. This finding is also supported by observations made in a study of 30 women with osteoporosis that were investigated for magnesium deficiency using a magnesium tolerance test [26]. The subjects were divided into three groups: ten with vitamin D insufficiency (low vitamin D and raised PTH); ten with functional hypoparathyroidism (low vitamin D and low/low normal PTH); and ten who were vitamin D replete (normal vitamin D and normal PTH). All ten subjects with functional hypoparathyroidism were found to be magnesium deficient; five magnesium-deficient and five magnesium-insufficient patients were found in the subjects with vitamin D insufficiency, and only one magnesium-deficient and four magnesium-insufficient patients were found in the vitamin D replete group. Furthermore, intravenous magnesium infusion led to a significant rise in PTH in the group with functional hypoparathyroidism and a reduction in PTH in the subjects with vitamin D insufficiency.

A number of previous studies have examined the associations between magnesium intake with risk of stroke [42,43] and coronary heart disease [43,44], however, the results have been inconsistent in these previous studies. Two meta-analyses of prospective studies found that magnesium intake was related to a significantly reduced risk of stroke [42,43]. However, the inverse association was weak (an 8% reduction in risk per 100 mg magnesium/day increment). Likewise, a previous meta-analysis found that magnesium intake was non-significantly inversely associated with coronary heart disease with a pooled RR (95% confidence intervals (CIs)) of 0.86 (0.67 to 1.10) for the highest intake versus the lowest intake category [43]. In the current study, we found that the HR (95% CIs) for risk of mortality due to cardiovascular disease was 0.88 (0.61 to 1.26) for the highest quartile intake of magnesium versus the lowest quartile intake, which is very close to that in previous studies. Several previous studies have also evaluated the associations between magnesium intake and risk of colorectal cancer and results have also not been consistent [45]. A very recent meta-analysis found that every 100 mg/day increase in magnesium intake was related to 12% reduced risk of colorectal cancer (RR: 0.88; 95% CI 0.81 to 0.97). In the current study, we found that the HR (95% CIs) for risk of mortality due to colorectal cancer was 0.76 (0.15 to 3.86) for the highest quartile intake of magnesium versus the lowest quartile intake, which is also consistent with those from previous cohort studies. The significant interaction between serum vitamin D and magnesium intake in relation to mortality for cardiovascular disease and colorectal cancer may also explain some inconsistencies in previous studies which examined the associations of magnesium intake alone with risk of cardiovascular disease and colorectal cancer.

A strength of our study is that it is based on NHANES, a population-based study with nationally representative samples. As with all prevalent case–control studies, one concern is that the temporal sequence may not be clear. However, it is unlikely that serum vitamin D status led to high or low intake of magnesium or vitamin D. Furthermore, the analysis of data from the NHANES III, a population-based prospective cohort, provides additional indications that magnesium interacts with vitamin D in relation to mortality. It is possible that people who consumed a high level of magnesium may also have a healthy lifestyle (for example, more physical activity and higher proportion of dietary supplement users). Supplement users may consume a high level of vitamin D from supplements while outdoor physical activity is related to an increasing production of 25(OH)D from sunlight exposure. We have adjusted for physical activity and intake of vitamin D from both diet and supplement in all analyses. Furthermore, we have conducted stratified analyses by physical activity and use of supplement. We found, unlike intake of magnesium, physical activity and use of supplements did not significantly or marginally significantly modify the association between serum 25(OH)D and mortality. Finally, although multiple 24-h dietary recalls are used as a gold standard measure in nutritional epidemiologic studies, a one-time 24-h dietary recall may not capture long-term dietary exposure. However, similar to dietary vitamin D and supplemental vitamin D intake, we found both dietary intake of magnesium (based on 24-h dietary recall) and magnesium from supplementation intake (derived from 30-day supplement questionnaire) significantly contributed to serum vitamin D status. Moreover, total intake of magnesium significantly interacted with intake of vitamin D in relation to both magnesium deficiency and insufficiency while total intake of magnesium modified the association between serum vitamin D and mortality. Since inter-day variation in magnesium intake is random, any residual inter-day variation may lead to non-differential misclassification, which usually biases the result to the null. Thus, the true associations of magnesium intake with vitamin D status risk may be stronger than those we observed. Our findings are not only biologically plausible, but also remarkably consistent, indicating our findings may not be solely due to chance. In the current study, we did not have data for other parameters of the vitamin D/PTH axis (that is, serum PTH and 1,25-dihydroxyvitamin D), which may indeed be affected by magnesium status. Future studies are warranted to examine if low magnesium status using dietary and body status of magnesium affected both PTH levels and 1,25-dihydroxyvitamin D because this could serve as one mechanism for the observed interaction between serum 25(OH)D and magnesium intake on mortality.

## Conclusions

Our preliminary findings indicate it is possible that magnesium intake alone or its interaction with vitamin D intake may contribute to vitamin D status. The associations between serum 25(OH)D and risk of mortality may be modified by the intake level of magnesium. Future studies, including cohort studies and clinical trials, are necessary to confirm the findings.

## Abbreviations

25(OH)D: 25-Hydroxyvitamin D; 95% CI: 95% confidence interval; HR: Hazard ratio; IOM: Institute of Medicine; NDI: National death index; NHANES: National Health and Nutrition Examination Survey; OR: Odds ratios; PTH: Parathyroid hormone; UVB: Ultraviolet-B; VDBP: Vitamin D binding protein.

## Competing interests

The authors declare that they have no competing interests.

## Authors’ contributions

XD and QD designed the current study and drafted the manuscript. XD carried out the statistical analysis. YS, JEM, LBS, MJS, RMN, DLS, and QD contributed to study design, data interpretation, and the critical review of the paper. All authors read and approved the final manuscript.

## Pre-publication history

The pre-publication history for this paper can be accessed here:

http://www.biomedcentral.com/1741-7015/11/187/prepub
